# A three-dimensional hybrid pacemaker electrode seamlessly integrates into engineered, functional human cardiac tissue *in vitro*

**DOI:** 10.1038/s41598-018-32790-8

**Published:** 2018-09-28

**Authors:** Tobias Weigel, Tobias Schmitz, Tobias Pfister, Sabine Gaetzner, Maren Jannasch, Reem Al-Hijailan, Sebastian Schürlein, Salwa Suliman, Kamal Mustafa, Jan Hansmann

**Affiliations:** 1 University Hospital Würzburg, Department Tissue Engineering and Regenerative Medicine (TERM), Röntgenring 11, 97070 Würzburg, Germany; 2 Fraunhofer Institute for Silicate Research, Neunerplatz 2, 97082 Würzburg, Germany; 3 King Faisal Hospital and research center, Cell Biology Department, research center, P:O Box, 3354 Mbc03, Riyadh, 11211 Saudi Arabia; 4 Department of Clinical Dentistry, Center of Clinical Dental Research, University of Bergen, Årstadveien 19, 5009 Bergen, Norway

## Abstract

Pacemaker systems are an essential tool for the treatment of cardiovascular diseases. However, the immune system’s natural response to a foreign body results in the encapsulation of a pacemaker electrode and an impaired energy efficiency by increasing the excitation threshold. The integration of the electrode into the tissue is affected by implant properties such as size, mechanical flexibility, shape, and dimensionality. Three-dimensional, tissue-like electrode scaffolds render an alternative to currently used planar metal electrodes. Based on a modified electrospinning process and a high temperature treatment, a conductive, porous fiber scaffold was fabricated. The electrical and immunological properties of this 3D electrode were compared to 2D TiN electrodes. An increased surface of the fiber electrode compared to the planar 2D electrode, showed an enhanced electrical performance. Moreover, the migration of cells into the 3D construct was observed and a lower inflammatory response was induced. After early and late *in vivo* host response evaluation subcutaneously, the 3D fiber scaffold showed no adverse foreign body response. By embedding the 3D fiber scaffold in human cardiomyocytes, a tissue-electrode hybrid was generated that facilitates a high regenerative capacity and a low risk of fibrosis. This hybrid was implanted onto a spontaneously beating, tissue-engineered human cardiac patch to investigate if a seamless electronic-tissue interface is generated. The fusion of this hybrid electrode with a cardiac patch resulted in a mechanical stable and electrical excitable unit. Thereby, the feasibility of a seamless tissue-electrode interface was proven.

## Introduction

By delivering small bursts of electrical energy, pacemakers control the beating frequency of the heart and save millions of peoples’ live every year^1^. A robust pacemaker performance, however, requires a permanent electrical contact of the electrode to the cardiac muscle. To remain mechanically attached, electrodes have either a hook or a screw that are embedded in scar tissue following implantation. If extensive formation of fibrotic tissue at the stimulation site occurs, the excitation threshold increases and the energy efficiency of the pacemaker system is impaired^2^. Thus, the response of the tissue to the electrode reduces the battery life span.

The formation of the fibrotic capsule around the electrode is a result of the innate immune system’s reaction to a foreign body^3^. This foreign body reaction is guided by material-tissue interactions. Hereby, it has been demonstrated that the implant properties strongly influence the intensity of the body’s defense mechanism^4^. For example, different structures, sizes and shapes of implants, like fibrous, porous or dense, manufactured from the same material result in a varying biocompatibility *in vivo*^5^. Moreover, a suitable material stiffness has been identified as a crucial parameter to improve the long-term response to an implant. Compared to stiff electrodes, flexible electrodes reduce micro-motion-induced mechanical trauma, which enhances the life span of the electrode-tissue interface^6^. In addition to size, shape, and stiffness, also the dimensionality of the implant, meaning the possibility for cell migration, affects the intensity of the foreign body response. For example, the implantation of an interconnected porous non-conductive scaffold in various organs fostered the integration of cells and the formation of functional tissue within the implant. Thereby, the foreign body reaction was dramatically reduced^7,8^. However, the biomaterials used in these studies focused on improving cell integration and did not address electrical properties as required for electrodes. To generate an electrode that can be infiltrated by cells, the surface of a conductive 2D material can be coated with a hydrogel that includes partial-electrical conductive polymers^9^. The hydrogel layer provides a 3D microenvironment and improves cell migration. The hydrogel renders a transition zone between the host’s tissue and the electrode. The electrode, however, is still a rigid planar surface. As an alternative to currently used planar metal and hydrogel-coated electrodes, a porous and conductive biomimetic scaffold was facilitated by combining electrospinning with a high temperature process^10^. The resulting scaffold showed a low electrical impedance and a high mechanical flexibility. In contrast to a standard electrospinning process, the adapted technology allowed to increase the mesh opening size as well as the porosity of the spun scaffolds. With pore sizes up 70 µm in height and several 100 µm in length as well as the extended mesh openings with sizes up to 60 µm², a biomimetic matrix-like structure is generated, which supports cell migration and ingrowth. The culture of cardiomyocytes derived from human induced pluripotent stem cells (hiPSC-CM) on this scaffold, revealed the formation of spontaneously beating cell clusters throughout the scaffold. Based on the evaluation of the electrical and mechanical scaffold properties as well as on the proven compatibility of the 3D scaffold with cardiac tissue, the feasibility of a three-dimensional, biomimetic matrix-like electrode scaffold was demonstrated. This research constituted the basis for the study presented here, where initially, the properties of the porous electrode scaffold are compared to a current high-performance pacemaker material (TiN). Furthermore, the progression on the development of a tissue-electrode hybrid composed of autologous stem-cell-derived tissue and conductive fibers was envisioned. In addition to a seamless integration of the electrode into the host’s tissue, a reduced foreign body reaction might be accomplished due to the decoration of the foreign body with host’s cardiomyocytes. Such a tissue-electrode hybrid also supports the regenerative capacity of the target tissue and foster pro-healing processes. This supportive feature is especially relevant for tissues with a relative low regenerative capacity and high risk of fibrosis - like heart tissue. However, establishing an electro-spun electrode completely covered by cardiac cells was not accomplished, yet. Additionally, it is unclear whether the synchronization of such a construct with the host tissue is possible. Thus, to assess the concept of a tissue-electrode hybrid, the functional integration of the hybrid into the host’s tissue need to be demonstrated.

The gold standard for the characterization of an implant are animal studies [ISO 10993-6:2007; www.iso.org], allowing to assess the long-term response of the tissue to an implant. The use of personalized induced pluripotent stem cells, as a major component of a tissue-electrode hybrid, however, hampers following the standard procedure. For example, for most species, suitable induced pluripotent stem cell lines are unavailable. Complimentary to animal tests, distinct mechanism of the organism’s response to an implant can be investigated *in vitro*. For example, an adapted monocyte activation assay based on human whole blood facilitates the detection of pyrogenic contaminations on implants^11^. Moreover, mechanistic *in vitro* models enable the ranking of biomaterials concerning their inflammatory potential^4^, and the macrophage-modulated recruitment of fibroblasts to the implant site as well as the induced tissue remodeling^3^. Although these *in vitro* assays facilitate to forecast an electrode’s potential to induce a foreign body response, the ingrowth of a hybrid electrode, which is composed of host cells and conductive leads, as well as the stimulation efficacy are still needed to be assessed using three-dimensional cardiac tissue. The generation of a vascularized human cardiac patch based on a biological scaffold has already been shown^12^. During *in vitro* culture up to four months in a bioreactor, the tissue maintained physiological characteristics, such as a spontaneous beating frequency of 1 Hz, and sensitively responded to drugs and external electrical stimulation. For the generation of the cardiac patch, human hiPSC-CM, human endothelial cells, human mesenchymal stem cells as well as human fibroblasts were seeded into a biological scaffold. The use of human cells, the physiological characteristics, and the possibility to perform long-term studies make the cardiac patch a suitable tool for the assessment of cardiac electrodes.

**Figure 1:**
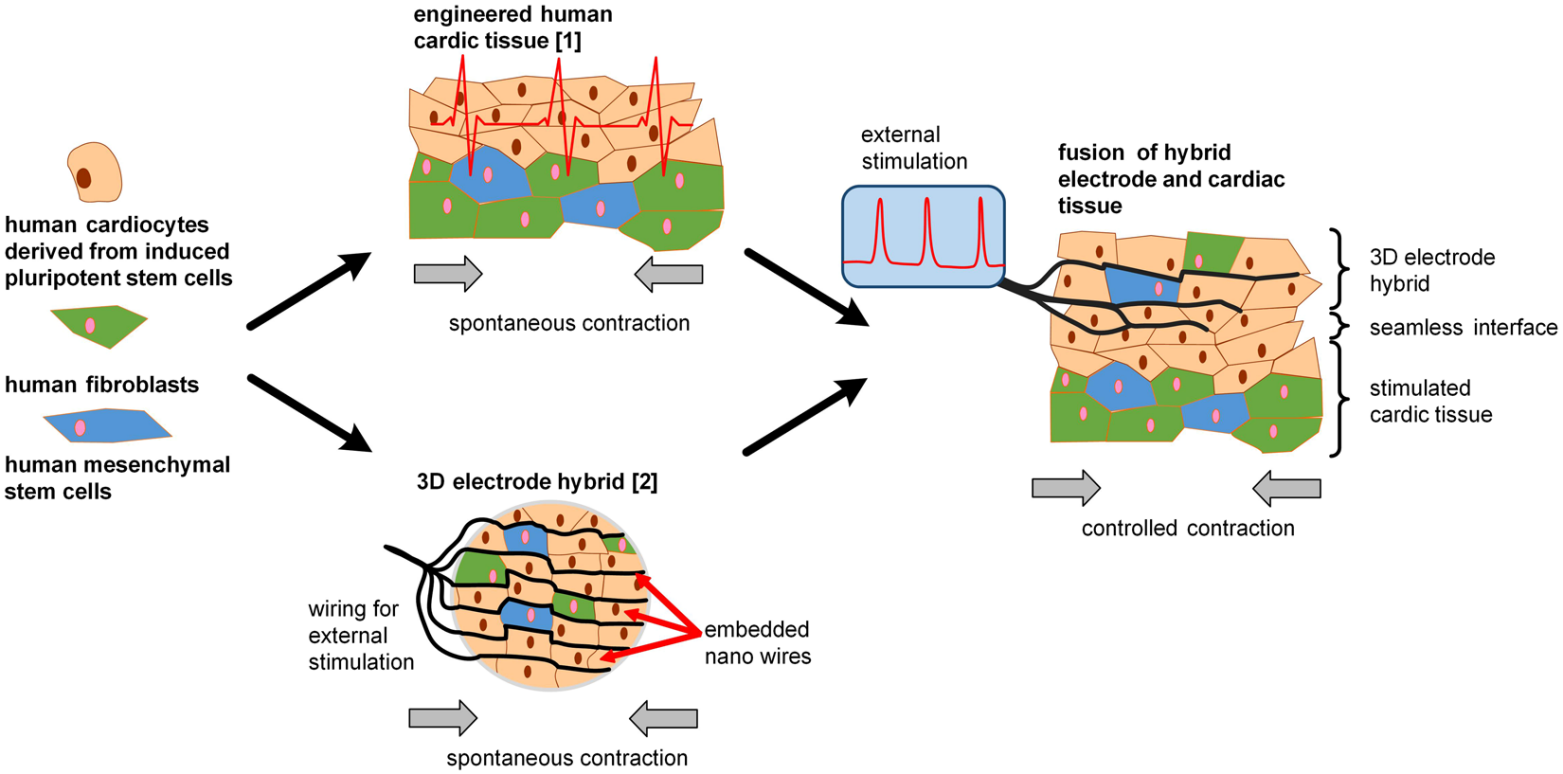
Assessment of an electrode hybrid composed of conductive fibers and human tissue. A functional, spontaneously contracting human cardiac tissue based on human induced pluripotent stem cells, fibroblasts, and mesenchymal stem cells was engineered to allow the characterization of pacemaker electrodes in a realistic microenvironment. As an alternative to current planar pacemaker electrodes, an electro-spun, porous 3D fiber electrode was decorated with human induced pluripotent stem cells, human fibroblasts, and human mesenchymal stem cells. Thereby, a spontaneously beating hybrid electrode was generated. The hybrid electrode exhibited characteristics comparable to cardiac tissue. When culturing both systems together, a seamless, mechanically robust integration of the 3D electrode into the cardiac patch was observed. Moreover, external stimulation through the fibers was possible.

Overcoming the limitations of current pacemaker electrodes constitutes a new frontier in material development: to optimize the energy efficiency of a pacemaker by achieving a physiological ingrowth of the electrode into the host’s tissue. This study aims to optimize material characteristics to control the interaction between the tissue and a pacing electrode. Therefore, a biomimetic electrode approach was harnessed. Porous fiber electrodes were transformed in tissue-electrode hybrid systems comprising conductive leads embedded in functional cardiac tissue (Fig. 1). Following maturation, the hybrid electrodes were implanted onto engineered human cardiac tissue. After a culture of four weeks, ingrowth, electrical synchronization, and electrode functionality were assessed. Hereby, the formation of a seamless electrode-tissue interface was detected.

## Material and Methods

### Ethical clearance statement

Primary human cells were isolated from biopsies, e.g. blood or foreskin. Cell isolation was approved by the ethical commission of the University of Wuerzburg (vote 182/10). Informed and written consent from either the patient or from the next of kin, caretakers, or guardians was obtained if the patient was underage. Experiments were conducted in compliance with the rules for investigation on human subjects, as defined in the Declaration of Helsinki.

Animal experiments were approved by the Norwegian Animal Research Authority and conducted in strict accordance with the European Convention for the Protection of Vertebrates used for Scientific Purposes (FOTS no. 12936).

### Generation of conductive porous fiber scaffolds

Porous carbon and nonporous fiber scaffolds that served as the conductive leads in the hybrid electrode were manufactured by a modified electrospinning process according to Weigel *et al*.^10^. Briefly, sodium chloride (NaCl)-particles with sizes between 70 and 125 µm were embedded in the fiber scaffold during electro-spinning of a 12 w/w% polyacrlonitrile (PAN)/N,N-dimethylformamide (DMF) solution (all chemicals purchased from Sigma-Aldrich, Schnelldorf, Germany). During two heating processes at 250 °C (air) and 1000 °C (Argon), the polymer fibers were carbonized. Additionally, the NaCl-particles melted and evaporated, resulting in a porous and conductive scaffold.

### Generation of TiN Layers

The Ti/TiN coatings were produced by physical vapor deposition on cleaned glass bowls (Brandt, Wertheim, Germany) and Ti foils (Goodfellow GmbH, Friedberg, Germany). First, pure Ti was deposited using a Ti target with argon as process gas. Afterwards, by adding nitrogen to the argon atmosphere, TiN coatings were deposited on the layer of pure Ti. Prior to cell tests, TiN samples were incubated in an ultrasonic bath with deionized water for 30 min, subsequently in 70% ethanol for 15 min, and finally autoclaved.

### Electrical characterization

Impedance spectroscopy and cyclic voltammetry (CV) were measured with a potentiostat PGSTST204 (Metrohm Autolab, Utrecht, Netherlands) in PBS^−^ as electrolyte. A four-electrode setup was used with a glassy carbon rod as counter electrode. Therefore, all samples were cut to size of 1.25 cm². The frequency range of the impedance measurement ranged from 0.1 to 10000 Hz with a voltage amplitude of 10 mV. The CV measurements were carried out for voltages between −1 and 1 V vs Ag/AgCl with a step size of 0.03 V/s.

To measure the formation of the bias voltage, samples with a surface area of 1.25 cm² were treated with electrical pulse strength (Stimulator HC FE155, AD Instruments, Spechbach, Germany) of 50 mA and a pulse width of 0.1 ms in Dulbecco’s Modified Eagle Medium (DMEM) as electrolyte. The resulting voltage between the tested electrode surface and the glassy carbon counter electrode (PowerLab 4/35, AD Instruments) was measured. Pacing with a pulse frequency of 1 and 2 Hz was applied for 60 s.

### Infiltration of 3D electrodes with hdF

Human dermal fibroblasts (hdF) were isolated from the dermal part of a human foreskin as previously described^13^. The cells were cultured in DMEM with 10% FCS (Gibco, Carlsbad, USA). For the infiltration experiments, the porous fiber scaffold was mounted in a cell crown with 1.1 cm² surface area^14^. The fibers were wetted with sterile DI-water, washed with culture medium, and subsequently seeded with hdF in a density of 15000 cell/cm². After 10 weeks of culture the scaffolds were prepared for SEM analysis. Therefore, the samples were fixed in 4% paraformaldehyde solution (Carl Roth, Karlsruhe, Germany) and dehydrated in an ascending acetone (Carl Roth) series. Afterwards, samples were dried by critical point drying (CPD 030, BAL-TEC, Liechtenstein) and finally sputtered with a 1 nm layer of platinum (EM ACE600, LEICA, Wetzlar, Germany). SEM analysis was performed with a Supra25 (Zeiss, Oberkochen, Germany), using the inLens SE1 detector and an accelerating voltage of 2 kV.

For the generation of the pseudo color pictures, a background picture containing the cellular part was produced by ImageJ. In the first step a general background image from the original image was generated by the ImageJ tool “Subtract Background” with a radius of 10 pixels. In the next step, this general background image was subtracted from the original image. This generated an image, which mainly contains the bright fiber structures. When subtracting these fiber structures form the general background image, a cellular background image was obtained. The final pseudo color picture was produced by an overlay of the original picture in gray and the cellular background image in red. Therefore, the intensity of the cellular background image was reduced to prevent a covering of the structural information of the original image.

### Characterization of material-induced inflammatory response

M2-like macrophages haven proven as a suitable cell type to assess the inflammatory response of a material^3,4^. The isolation of monocytes and the pre-polarization to M2-phenotype was performed by a protocol published by Jannasch *et al*.^3^. Briefly, mononuclear cells were isolated by ficoll gradient centrifugation (GE Healthcare, Freiburg, Germany) from peripheral whole blood. The monocytes were purified by negative magnetic cell separation (MiltenyiBiotec, Bergisch Gladbach, Germany) and cultured in RPMI GlutaMax (Gibco) supplemented with 10% FSC. Differentiation to macrophages was performed by adding 40 ng per ml recombinant human M-CSF (Peprotech, New Jersey, United States) for 6 days.

For M2-macrophage monocultures, the fiber scaffold was clamped in a 3D-printed cell crown with closed bottom and an area of 1 cm². For the TiN-samples, glass petri dishes with 7 cm² were sputter coated described in 2.3. The macrophages were seeded with a density of 1.2 × 10^5^ cells/cm² and a volume-to-surface ratio of 440 µl medium/cm². The culture was performed in RPMI medium (Thermo Fisher Scientific, Carlsbad, USA) containing 10% FCS for 48 h.

The secretion of human IL-1ß, IL-6, IL-8, IL-10, IL-12 and TNF-α in cell culture supernatant were measured by human Inflammatory Cytokine CBA (551811, BD Biosciences, Heidelberg, Germany) according to manufacturer’s protocol. Analysis was performed with FACS Calibur (BD Biosciences) and data was processed using FCAP Array Software 3.0 (BD Biosciences).

For co-cultures, M2-macrophages were combined with the fibroblast cell line SCRC-1041 (ATCC, Manassas, Virginia, USA). Fiber scaffolds as well as TiN-coated titanium foils were clamped in a 3D-printed cell crown with open bottom and an area of 1 cm². To ensure sufficient nutrient exchange, the cell crown walls of the TiN samples were perforated using a 0.5 mm driller. The seeded cell density amounts to the M2-macropages 3 × 10^4^ cell/cm² and 1.5 × 10^4^ cells/cm² for the SCRC-1041, a ratio of 2:1. The culture was performed in RPMI medium containing 10% FCS, 1 mM Na-pyruvate as well as 100 µM L-ascorbic acid 2-phosphate sesquimagnesium salt hydrate for 24 days with medium changes 3 times per week. After 4 weeks of culture, the cells were lysed by incubating the samples two times for 15 min in 80 mmol/L sodium desoxycholate (Sigma-Aldrich) and washed three times in PBS^−^. SEM preparation was performed as described in 2.5.

### Host tissue response assessed *in vivo*

Eleven female Wistar rats (6–8 weeks old) were anaesthetised using Isoflurane (Isoba® vet) (Schering Plough, Kenilworth, NJ, USA) before two incisions (~2 cm) were made on their back. One incision was made between the upper limbs and another between the lower limbs and a pouch was dissected on each side. Scaffolds with the size of 1 cm × 1 cm were implanted into each rat and the different groups were distributed randomly. Wounds were sutured with Vicryl 4-0 and the animals were given Buprenorphine (Temgesic® 0.3 mg/kg) subcutaneously as analgesic. Animals were sacrificed with CO_2_ overdose 4 and 30 days after implantation (at least 5 animals per time point). The samples were harvested and fixed in 4% paraformaldehyde before being processed and paraffin embedded. Sections of 3–4 μm were stained with hematoxylin and eosin.

### Generation of the hybrid electrode

To seed the conductive electrode mesh with cells, scaffolds were clamped in a custom 3D-printed cell crown with a cell culture surface of 1.1 cm². For the electrical connection, a carbon fiber stripe of 2 cm in length and 0.5 cm width was prepared on one side of the scaffold, and was embedded in orthopedic silicone (Biopor AB, Dreve Otoplastik GmbH, Unna, Germany). Prior to cell seeding, scaffold-cell-crown constructs were sterilized by autoclaving and placed in a 6-well plate (TPP, Trasadingen, Switzerland). HiPSC-CM were differentiated from the iPS cell line IMR 90-4 by the differentiation protocol of Kadari *et al*.^15^. The reported purity of cardiomyocyte-like cells was up to 95%. Following, hiPSC-CM, human mesenchymal stem cells (MSC), and hdF were mixed in the ratio 1.5/1/0.5 and seeded with a total cell density of 2 × 10^6^ cells/cm² on the 3D electrode scaffolds. Medium change was performed 3 times a week for a total duration of 4 weeks. For the entire cell culture duration, hiPSC-CM medium described by Kadari *et al*.^15^ was used. During culture, the hybrid electrode was not electrically stimulated.

### Electrophysiology of the hybrid electrode

The electrophysiological measurements were performed by a multi electrode array (MEA, Multi Channel Systems MCS GmbH, Reutlingen, Germany). Therefore, hybrid electrodes were cut in pieces of approximately 4 × 4 mm and placed on an 8 × 8 MEA-chip (Qwane Biosciences SA, Lausanne, Switzerland) without corner electrodes. The electrodes of the MEA-chip were built as tip-shaped 3D platinum electrodes with a diameter of 30 µm, a height of 50–70 µm, a spacing of 200 µm and an impedance of 450–650 kΩ.

### Generation of the human cardiac tissue

To assess the ingrowth of the 3D electrode systems, human tissue-engineered cardiac patches were used. These test systems were based on a previously published protocol^12^. Briefly, a decellularized part of a porcine jejunum with removed villi and without serosa was used as a biological scaffold^16^. This matrix was cut in pieces of approximately 2.5 to 2.5 cm. Scaffold pieces were fixed using a cell crown (stainless steel) according to Moll *et al*.^14^. Following, cells were seeded on the matrix. The seeded cell suspension consisted of hiPSC-CM, human MSC, and hdF in the ratio of 1.5/1/0.5. Medium was exchange 3 times a week over a time period of 4 weeks. During culture, the medium for the hiPSC-CM described by Kadari *et al*. was used^15^ and no electrical stimulation was applied.

### Ingrowth experiments

The hybrid electrode composed of conductive leads and human tissue was detached from the cell crown and cut into a square of about 0.7 cm edge length. The electrode was turned upside down and directly placed in the cell crown of the cardiac tissue (2.9). The wiring for external stimulation pointed upwards out of the cell crown. The cardiac tissue partly covered by the hybrid electrode was cultured for 4 weeks with medium change 3 times a week. Culture medium was the same as in 2.9. During culture, the stacked cardiac patch and hybrid electrode were not electrically stimulated.

### Functional testing of the cardiac patch/hybrid electrode construct

The construct composed of electrode and cardiac patch was sectioned to form a 0.5 mm width stripe, containing the silicone embedded electrical connection. Both ends of the patch were mounted to a force transducer in an organ bath (DMT Myograph PL3504B16, AD Instruments). After 30 min, the clamped patch was tensed until the contraction signal could be recorded. For the electrical stimulation, a stimulator (Stimulator HC FE155, AD Instruments) was connected to the silicone-embedded carbon fiber wiring for external stimulation. The applied monophasic electrical pulses had a pulse width of 0.1 ms and a current of 50 mA.

To measure the mechanical stability of the electrode-tissue interface, force transducers of the organ were connected to the silicone embedded carbon fibers and the cardiac tissue. Following, the distance between the force transducers was continuously increased until the cellular connection between the electrode and patch broke. The maximum applied force was used to assess the mechanical stability of the electrode-tissue interface.

### Immunohistological staining and microscopy

Samples were fixed in 4% paraformaldehyde solution (Histofix, Carl Roth, Rothenfels, Germany). After permeabilization with 0,1% Triton X-100 (Sigma-Aldrich) for 10 min, non-specific-binding was inhibited by incubation in 5% donkey serum (species of the secondary antibody) for 20 min. Incubation of the primary antibody was carried out at 4 °C over night with following antibodies and dilutions: 1:1000 for anti-cardiac Troponin T (cTnT) (rabbit, Sigma-Aldrich), 1:2000 for anti-Vimentin (mouse, Abcam, Cambridge, UK), 1:100 for anti-α-Actinin (mouse, Sigma-Aldrich), 1:100 for anti-CD90 (rabbit, Abcam) and 1:100 for anti-CD54/ICAM-1 (mouse, Invitrogen, Carlsbad, USA). Secondary antibodies were diluted 1:100, with alexa fluor 488 anti-mouse (donkey, Invitrogen) and alexa fluor anti-rabbit (donkey, Invitrogen). Phalloidin (Cell Signaling Technology, Cambridge, UK) staining was performed with a dilution of 1:40 in PBS^−^ containing 1% BSA. The incubation time was 20 min. Nuclei were stained with DAPI (Sigma-Aldrich). Confocal fluorescent images were captured with a confocal microscope (TCS SP8, Leica, Wetzlar, Germany). Visualization of the fibers was performed by confocal reflection (excitation 476 nm, detection at 474–487 nm).

### Statistical analysis

Data were expressed as the mean ± SD. Statistical analysis was carried out using an unpaired student’s t-test and ANOVA analysis of variance. A value of p < 0.05 was statistically significant.

## Results

**Figure 2:**
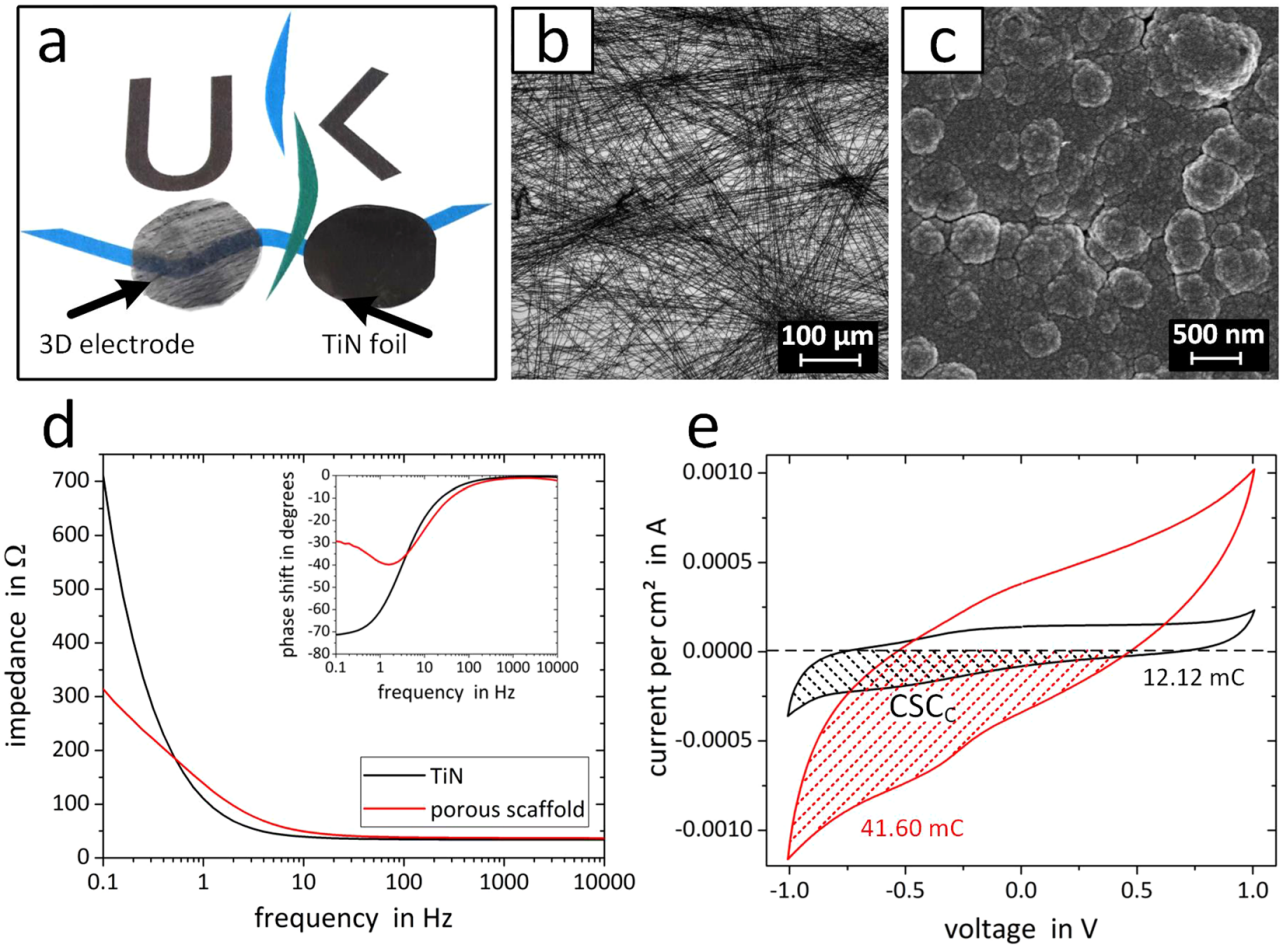
Comparison of the structural and electrical properties of a 3D carbon nano fiber scaffold and a 2D TiN surface. (**a**) Photo of the slightly translucent carbon fiber scaffold and a TiN coated Ti foil with diameters of 1.1 cm. (**b**) Light microscopy imaging visualizes the carbon fiber network with cell-infiltrateable mesh openings. (**c**) SEM imaging of the planar TiN surface revealed a nano-scale topography. (**d**) Impedance spectroscopy of both materials. The inset depicts the respective phase shift. (**e**) Cyclic voltametry measured in the potential window of −1.0 V and 1.0 V vs Ag/AgCl. The area between the negative current and the x-axis (hatched) describes the cathodal charge storage capacity (CSC_C_).

The development of biomimetic 3D hybrid electrode implants requires (I) a fibrous, flexible and conductive scaffold, (II) comparable electrical properties to current electrode materials, (III) reduced inflammatory properties, containing an active cell migration over a long time period and (IV) the generation of the functional target tissue to form a decorated electrode. While (I) was already discussed previously^10^, the electrical (II) and inflammatory (III) properties of the porous carbon fiber electrode were characterized in comparison to a current pacemaker electrode material. As the compatibility to cardiac cells was also confirmed previously, the formation of a functional tissue electrode (IV) with the ability for tissue integration and stimulation, was described in this study. To assess the electrical performance of the carbon fiber scaffold, 3D scaffolds were compared to 2D titanium nitride (TiN) layers (Fig. 2a). TiN represents a surface material currently used in modern pacemaker electrodes. Since the structure of the electro-spun 3D scaffold is a combination of highly-ordered and loose nano fiber arrangements, increased mesh openings, and a deployment of pores within the scaffold (Fig. 2b) were achieved. Whereas, the TiN reference material, exhibited a planar and smooth surface (Fig. 2c). Both materials showed comparable impedance properties (Fig. 2d). Only minor differences in the magnitude were detected for frequencies in the range between 0.9 Hz to 10000 Hz. Moreover, a slightly elevated impedance was found for the carbon fibers. In contrary, below 0.9 Hz, higher impedance values were detected for TiN, and the magnitude slope dropped more than two-times stronger compared to the porous scaffold. In the CV measurements, the porous fiber scaffold revealed an increased current flow compared to the TiN surface (Fig. 2e). Both materials, showed no adverse surface reactions in a selected voltage range of −1.0 V to 1.0 V. By integrating the negative current over time, the cathodal charge storage capacity (CSC_C_) was calculated^17^. Here, the CSC_C_ of the nano fiber scaffolds was about 3.5-times higher than the CSC_C_ of the 2D TiN layers. An increased charge capacity as well as trapped charges in the fiber structure formed a bias voltage on the electrode surface. Pacing for 1 min with 1 Hz resulted in a bias voltage of 0.122 ± 0.004 V (Supplementary Figure S1), while a pacing frequency of 2 Hz generated a bias voltage of 0.167 ± 0.005 V. Most striking, the TiN surfaces showed at least 3-times higher bias voltage compared to the here presented macro porous fiber electrodes.

**Figure 3:**
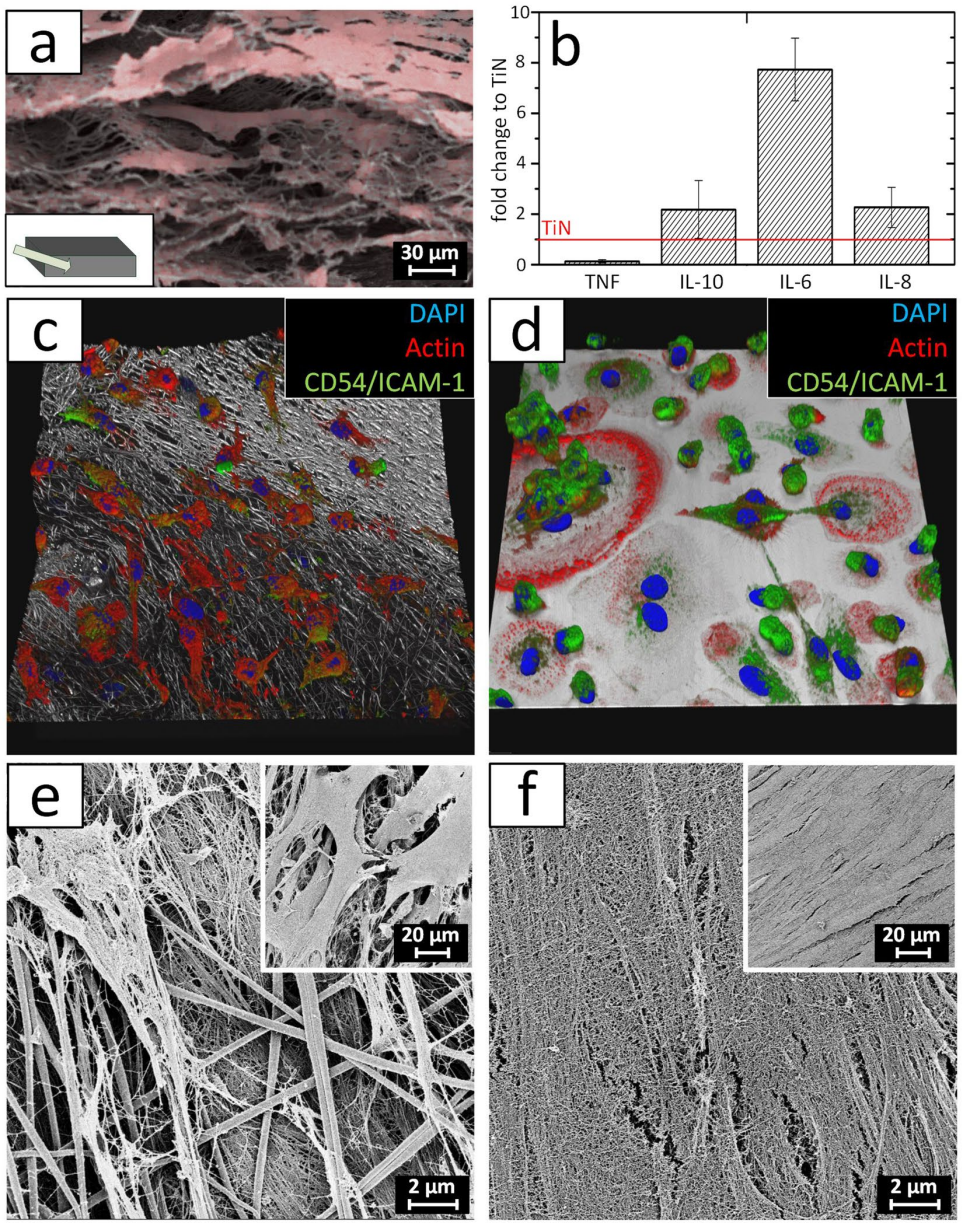
Cell integration and immunological properties of 2D and 3D electrodes. (**a**) False color SEM image showing a cross section of the porous fiber electrode (gray). The scaffold is completely infiltrated by hdF (red) after 10 weeks of culture. (**b**) Cytokine expression profile of M2-polarized macrophages cultured for 48 h on the 2D and 3D electrode. The values are normalized to the cytokine secretion measured for the planar TiN surface (N = 5). Confocal microscopy images of the M2 macrophages after 48 h on a (**c**) porous carbon fiber electrode and (**d**) a flat TiN surface. The actin skeleton is shown in red, CD54/ICAM-1 in green, and the electrode surface in gray. The image covers a section of 185 × 185 µm. SEM of a co-culture of M2 macrophages and SCRC-1041 fibroblasts after four weeks of incubation and following decellularization of the sample. (**e**) Between the fibers of the 3D scaffold, an additional protein network was established during culture. (**f**) The planar TiN electrode is completely covered with extracellular matrix. The inserts in (**e**) and (**f**) depict both electrodes prior to decellularization.

To assess whether the 3D electrode scaffold can be infiltrated by cells and integrated in to a tissue following implantation, human dermal fibroblasts (hdF) were cultured in the scaffold. After 10 weeks of culture, scanning electron microscopy (SEM) confirmed the infiltration of the hDF throughout the scaffold (Fig. 3a). The remodeling of the material and the deposition of extracellular matrix proteins is a crucial step during the foreign body immune reaction. An overshooting protein synthesis, leads to the formation of a fibrotic capsule that increases the excitation threshold. By the release of soluble factors, macrophages take a modulating function during the fibrotic response^3^. To compare the response of macrophages seeded in the 3D electrode or seeded on the 2D TiN surface, both materials were incubated with macrophages exhibiting M2-like phenotype. A material-dependent macrophage response was indicated by the measurement of cytokines levels that are known to play a role during fibrosis (Fig. 3b). The expression of IL-10, IL-6, and IL-8 were raised on the nano fiber scaffold, whereas almost none TNF was found in the supernatant of the cultured fibers. Differences were not significant (p > 0.05). In addition, microscopic analysis revealed material-dependent macrophage morphology. While cells cultured in the 3D scaffold were distributed throughout the fiber network (Fig. 3c, Supplementary video V1), cell clusters were detected on the 2D TiN samples (Fig. 3d, Supplementary video V2). Moreover, macrophages in the 3D network of conductive fibers showed a small, elongated morphology, whereas cells on the TiN layers had small and round as well as spreaded shape. Moreover, multinucleated cells were detected. In order to understand the macrophage-modulated deposition of extra cellular matrix proteins by fibroblasts, both cell types were incubated on both materials. This approach allowed identifying the effect of the electrode on the formation of a fibrotic capsule. When decellularizing the obtained tissues, deposited matrix fibers and free conductive carbon fibers were found in the 3D electrode (Fig. 3e). The fiber surface just exposes a rough surface in the size of a few nanometers, indicating a nonspecific protein adsorption from the medium during the 4 weeks of culture. In contrast, the 2D TiN surface was completely covered by a dense cellular and protein fiber network (Fig. 3f).

**Figure 4:**
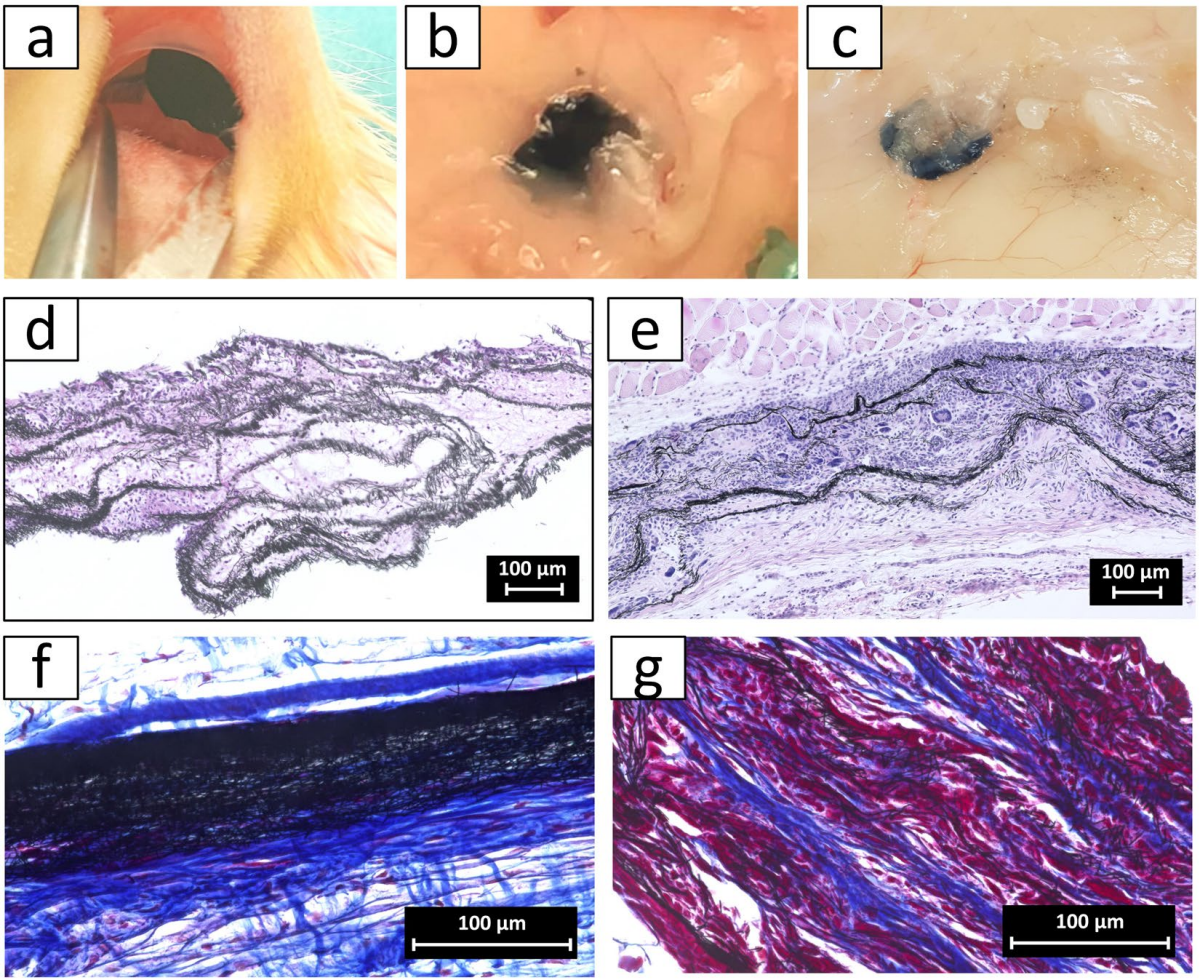
3D electrode scaffolds tested in a subcutaneous rat model. (**a**) Subcutaneous implantation of the scaffolds. Harvesting of the scaffolds and surrounding tissue after 4 days (**b**) and 30 days (**c**). H&E-staining of a 3D scaffold 4 days (**d**) and 30 days (**e**) post-implantation. Azan staining of 30-days implanted (**f**) dense and (**g**) porous carbon fiber scaffold. In contrast to the dense control group, the fibers of the porous scaffold are well embedded in the tissue.

Following the characterization of the scaffolds *in vitro*, the *in vivo* host response to the 3D fiber electrodes was investigated. The scaffolds were implanted subcutaneously in rats (Fig. 4a) and after implantation, the wounds healed normally at both time points. Animals observed up to 30 days showed no signs of systematic or neurological toxicity, based on scoring for lack of infection, total weight loss (>10%), hindered mobility and ungroomed fur. The cell infiltration and tissue integration was examined histologically after 4 and 30 days. During harvesting of the scaffolds, no adverse inflammatory reaction was detected macroscopically around the implants after 4 (Fig. 4b) and 30 days (Fig. 4c). The cross section at day 4 post-implantation (Fig. 4d,e) showed a rapid infiltration of fibrous tissue in the entire scaffold populated with polymorphnuclear cells, evident of early inflammatory response. Until day 30, the cellular content in the scaffold raised predominantly populated by fibroblasts and some foreign body giant cells could be seen within the scaffold (Fig. 4e). Regarding the capsule formation, a thin, non-continuous fibrous capsule (between 3–4 fibroblast layer thickness) was found around the implant. In summary, the scaffold was well integrated in the host’s tissue.

**Figure 5:**
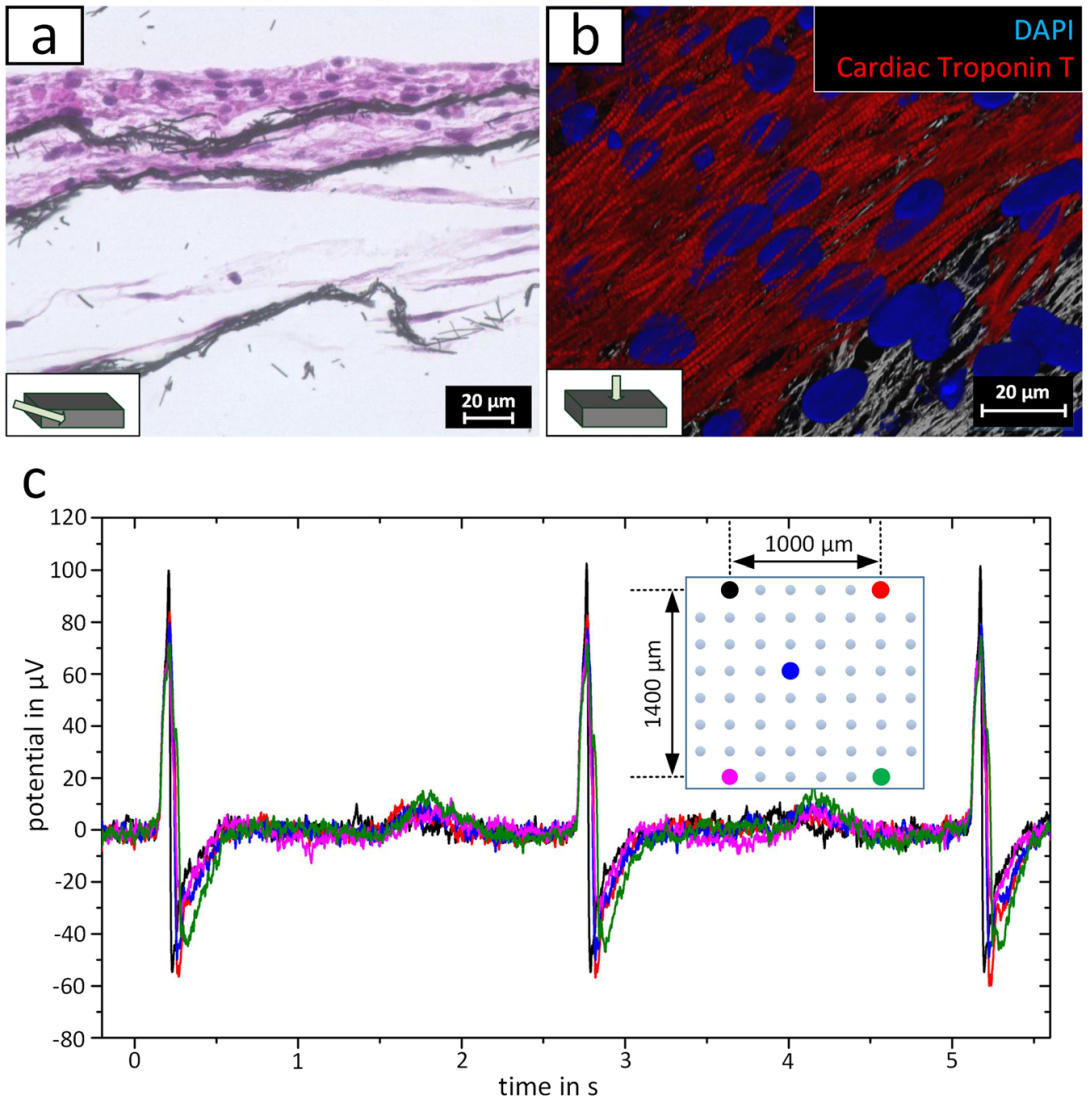
Hybrid cardiac electrode composed porous carbon nano fiber electrode and functional cardiac tissue after 4 weeks of culture. (**a**) Light microscopy image of the H&E stained cross section visualizing the porous electrode fiber bundles embedded in cellular tissue. (**b**) Immunoflourescent staining against cardiac Troponin T (red). Nuclei are shown in blue and the fiber electrode in gray. (**c**) Characterization of the electrical signal propagation in the hybrid electrode by MEA measurement. The different colors of the curves represent the position of the respective measuring electrode that is displayed in the inset.

The impact of the porosity of the scaffold was also detected when the collagen deposition around the scaffold was compared to a nonporous control. While a dense fibrous capsule with 5–9 layers was found for a nonporous 3D scaffold, the fibers of the porous scaffold were well-embedded in the tissue and only a weak fiber collagen deposition was detected. Due to the application as a pacemaker, the conductive 3D scaffolds were transformed into a tissue-electrode hybrid. Therefore, hiPSC-CM, hdFs, and hMSCs were seeded into the carbonized electro-spun scaffolds. After 2 to 3 days, spontaneously contracting cell aggregates were observed. In the following 4 weeks, cell aggregates spread throughout the scaffold, resulting in a synchronized contraction of the entire scaffold (Supplementary video V3). The contraction frequency of the hybrid electrode differed from model to model between 0.5 Hz and 1 Hz. Histological analysis demonstrated the formation of tissue layers with embedded carbon fibers (Fig. 5a). Due to the limited cell number, the scaffold was embedded up to the third fiber layer. The thickness of the cell layers ranged from a monolayer to around 10 cell layers, indicating that cell aggregates partially remained to some extent. Immunofluorescence microscopy of cardiac Troponin T (cTnT) confirmed the formation of muscle tissue inside the electrode scaffold (Fig. 5b). Except of a few regions, the expression of cTnT was distributed throughout the whole scaffold, and exhibited a locally oriented cellular structure (Supplementary Figure S2a). To track the fate of the hDFs and hMSCs populations, cross-sections were stained for alpha Actinin, CD90 and Vimentin (Supplementary Figure S3a–c). While alpha actinin was detected in all cell layers in a mostly unorganized manner, CD90 expression was limited to the lower tissue layers inside the scaffold. Vimentin-positive cells were found in the lower cell layers as well as on top of the tissue-electrode hybrid surface. This behaviour is similar to Schuerlein *et al*.^12^, where a vascularized cardiac patch was developed. Multi electrode array measurements visualized the electrophysiological properties of the seeded electrode scaffold (Fig. 5c). Thereby, the synchronized signals were recorded for all 60 electrode positions, with time shifts of 0.3–5.4 ms between the edge electrodes (Supplementary Figure S2b). Based on the distance between the electrode and the time shift between the recorded signals, a conduction velocity of 0.9 ± 1.1 m/s was calculated. This atypical signal conduction indicated that the electrical signal was transmitted throughout the fiber network.

**Figure 6:**
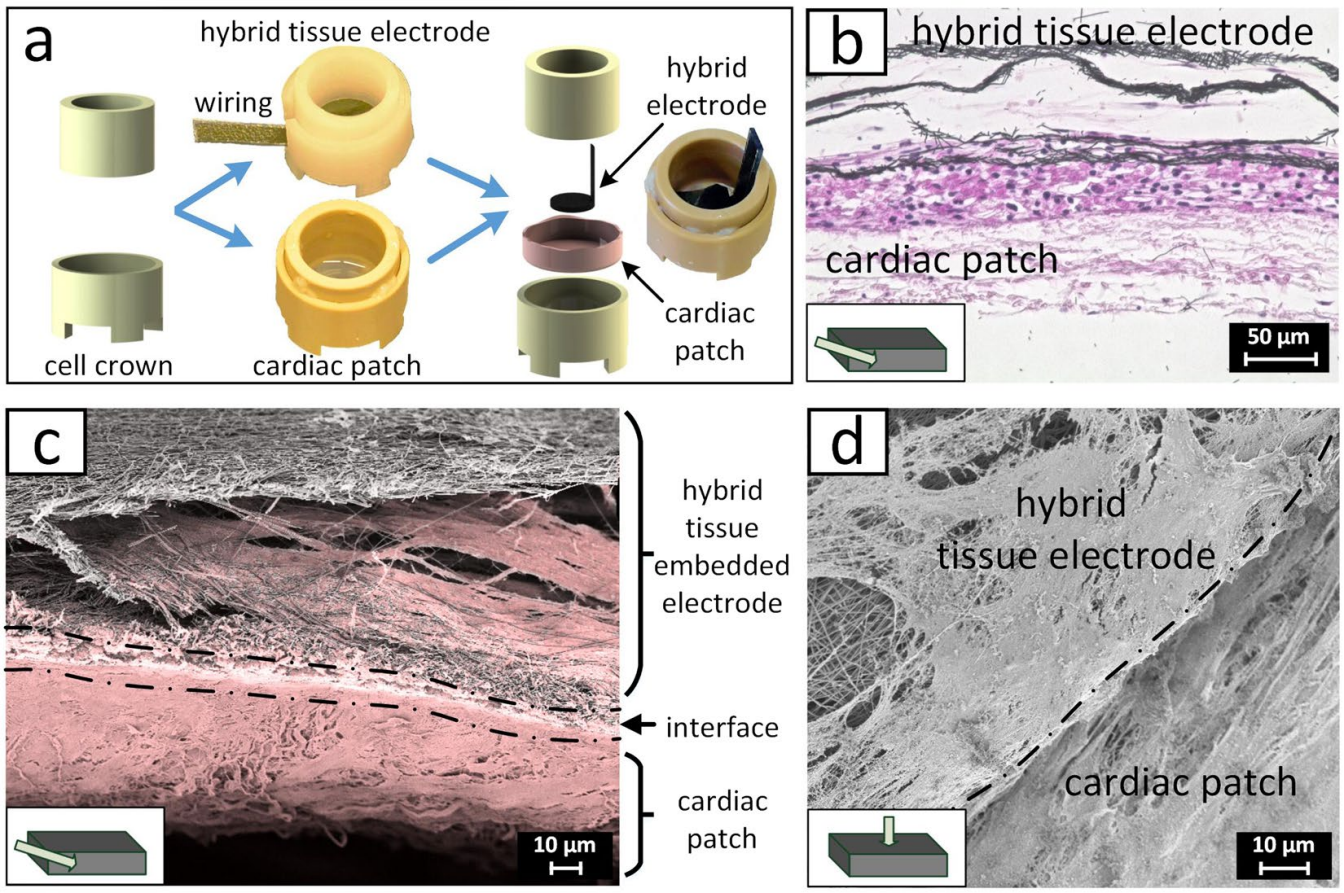
Ingrowth of the hybrid electrode into a cardiac muscle. (**a**) Detailed description of the experimental design. Cell crowns were applied to generate the hybrid electrode as well as the cardiac patch. For the ingrowth experiments, the hybrid electrode was transferred to the cardiac tissue. Therefore, the hybrid electrode was placed upside down on the cardiac patch and cultured for 4 weeks. (**b**) Light microscopy imaging of the H&E stained cross section showing the hybrid electrode that is fused with the cardiac patch. (**c**) False color SEM image showing the cross section of the electrode-tissue interface. Fibers are depicted in gray and cells as well as biological tissue in red. (**d**) SEM image of the electrode boundary and the underlying cardiac muscle.

A suitable tissue-electrode interface requires a seamless integration of the electrode into the target tissue without the formation of a fibrotic capsule in the proximity of the electrode. To test whether the tissue-electrode hybrid is able to fuse with cardiac tissue and to form an electrically synchronized unit, the spontaneously beating electrode system, was cut into patches of approximately 0.5 cm^2^, and was transferred onto a functional tissue-engineered cardiac patch (Fig. 6a). The spontaneous contraction of the engineered cardiac muscle can be seen in the Supplementary video V4. Like the hybrid electrode, the cardiac patch was generated with coculture of the same cells on a decellularized small intestine, resulting in a dense cellular layer on the rather cell free scaffold after 4 weeks. After additional 4 weeks of culture with the combined hybrid electrode and patch, histological analysis demonstrated that both components merged without a detectable interface region (Fig. 6b). The cellular cardiac tissues on the components fused together, forming a cellular unit between the electrode scaffold and the decellularized biological scaffold. In addition, underneath the cardiac muscle, the biological scaffold that served for the generation of the cardiac patch was observed. Immunofluorescent staining revealed comparable results regarding the fate of the hDF and hMSCs as found for the tissue-electrode hybrid (Supplementary Fig. S2d–f). The absence of a transition zone was confirmed by SEM (Fig. 6c). Hereby, a complete embedding of the first fiber layer into the cardiac construct was detected. Moreover, at the edge of the tissue-electrode hybrid, cells started to overgrow the electrode’s surface (Fig. 6d), indicating a strengthening of the mechanical robustness of the electrode-tissue interface and an ongoing integration process.

**Figure 7:**
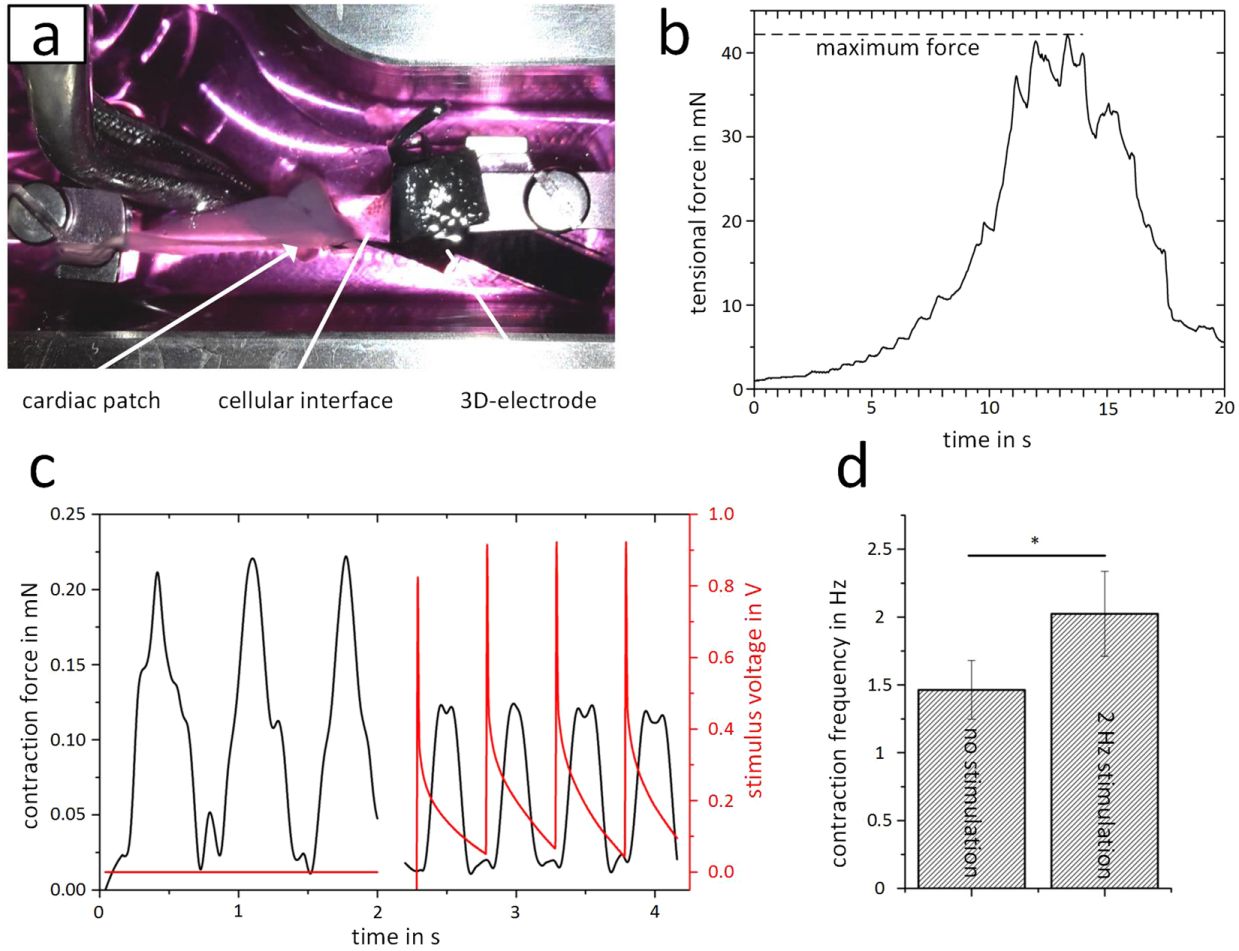
Functional testing of the hybrid electrode fused with the cardiac patch. (**a**) Photo of a tension experiment in an organ bath. The cardiac patch is clamped on the left side, while the silicone embedded wiring is clamped to a force transduce at the opposite side. The picture shows the electrode-tissue interface shortly before the complete disconnection. (**b**) Exemplary force curve, when continuously increasing the distance between the force transducer and the clamped tissue. The point of failure is shown in the maximum force. (**c**) Contraction measurement of the construct composed of the hybrid electrode embedded in the cardiac tissue. Without external stimulation, a spontaneous contraction with a frequency of approximately 1 Hz was detected. Under pacing, the contraction is synchronized to the external signal. (**d**) External stimulation with a 2 Hz signal results in an increased contraction frequency compared to the spontaneous beating. For the statistical analysis of the contraction frequency, 50 contractions of each construct (N = 3) and stimulation frequency were used.

The mechanical robustness of the interface was investigated by measuring the force required to detach the electrode fibers from the cardiac muscle (Fig. 7a), and the maximum detected pullout force was selected as criteria (Fig. 7b). Forces of 35.0 ± 13.0 mN were found after 4 weeks of culture. Due to an unknown cellular contact area between both components, the deployment of stress or tension is too inaccurate. Finally, the electrophysiological functionality of the interface was assessed by measuring the contraction force with or without pacing in an organ bath (Fig. 7c). In the absence of an external trigger signal, a spontaneous beating frequency of 1.46 ± 0.22 Hz was observed. When applying electrical pulses, the cardiac tissue contraction was able to follow a frequency up to 3 Hz. Figure 6d shows exemplarily the electrical pacing of 2 Hz, resulting in a contraction frequency of 2.02 ± 0.31 Hz.

## Discussion

To overcome limitations of pacemaker electrodes, the generation of a 3D tissue-electrode interface presents an alternative to currently used 2D systems^18^. In contrast to a planar surface, a 3D electrode facilitates a biomimetic microenvironment, which can be populated by cells more physiologically than a 2D surface. Moreover, the decoration of such a 3D electrode with autologous functional tissue might foster tissue ingrowth and finally the outcome of a patient’s therapy. Especially for tissues with a low regenerative potential such as the heart muscle, an electrode populated with autologous, tissue-specific cells might be beneficial. By using human hiPSC-CM, the feasibility of generating a functional and contracting tissue in a conductive porous scaffold was demonstrated. The obtained tissue-electrode hybrid furthermore integrated seamlessly into a spontaneously beating tissue-engineered cardiac muscle. Thereby, the feasibility of a novel approach for pacemaker electrodes was demonstrated.

A previously developed biocompatible electro-spun fiber scaffold rendered the basis for the hybrid electrode^10^. Compared to the planar TiN layers, the 3D carbon fiber electrode showed an improved electrical impedance for low frequencies. The good performance of the 3D electrodes can be explained by an increased contact area, which also resulted in higher charge storage capacities. In comparison to TiN standard electrodes, during pacing a lower bias voltage of the fiber electrodes reduces the susceptibility of tissue damage and unexpected signals. The fibers provide a structure that could be remodeled by fibroblasts, which also play a major role in the foreign body response. Following implantation, the long-term response to a material is modulated by macrophages^19^. *In vitro* culture of M2-like macrophages revealed differences in cell morphology between the 3D electrode and planar TiN surfaces. Due to the planar structure and the limited surface area on the TiN samples, cells started to form multi-nucleus cells on TiN. The fusion of M2 macrophages and the subsequent development to foreign body giant cells is described as one of the first steps towards the encapsulation of the electrode^3,19^. The fiber structure with adequate mesh openings provided a suitable inflammatory environment with a 3D morphology, where the pro-inflammatory TNF-α was reduced, and the anti-inflammatory IL-10 increased. Additionally, IL8, which induces chemotaxis and cell motility^4^, was raised in comparison to the planar TiN. When seeding macrophages and fibroblasts as main participants in the encapsulation process, the 3D electrode showed still a loose fiber network with the hint of protein adsorption on the fiber surface due to the formed surface roughness^20^. In contrast, the 2D surface was completely covered by a cellular/protein layer indicating the formation of a fibrotic capsule.

In addition to the characterization of the nano fiber electrode *in vitro*, this was supported with the evaluation of the early and late host response to the material in an established *in vivo* model to test biocompatibility of biomaterials. Thereby, effects such as protein adsorption or multi-cellular differentiation, which are absent in the *in vitro* setup, can be investigated. Already after 4 days, a high infiltration of host cells was detected confirming the infiltration assays performed *in vitro* and a normal early reaction to a foreign body implantation was found. After 30 days, cells continued to infiltrate, increasing in number with reduced matrix or collagen fibers in comparison to the early time point. A thin formation of a capsule was partially detected in the peripheries, which indicates the integration of the scaffold with the host tissues and a resolution of the foreign body reaction can be predicted. In comparison to other porous materials that are designed to reduce the foreign body response and the formation of a fibrous capsule, the carbon fiber mesh showed comparable host reactions^21^. Interestingly, the comparison of the nonporous and porous scaffold both composed of the same material revealed the impact of the scaffold porosity. As both scaffolds consisted of the same material, the detected effect can be correlated to the scaffold structure. Concluding, a fibrous capsule can be reduced, when a suitable electrode architecture is provided. Related to 2D electrodes with several materials^22^, the reduced capsule formation of the porous electrode implies a reduced foreign body reaction. In conclusion, the 3D nano fiber electrode integrates well into a host tissues without the induction of an adverse foreign body response.

After positive feedback regarding cell migration, possibility to seed cardiomyocytes^10^, macrophage interaction and fibrosis, the next step towards a hybrid electrode was the generation of cardiac tissue with embedded electrode nano fibers. The periodical contraction of the electrode as well as the extensive cTnT expression showing typical sarcomeres structures confirmed the generation of a functional electrode hybrid composed of conductive leads and cardiac muscle tissue. Comparison of nano fibers and cardiac cell orientation revealed a correlation between both. The parallel fibers between the fiber nodes promoted the cardiac cell orientation and accumulation. Regions lacking cTnT were likely located on the fiber nodes and may rather serve as access for cells to migrate into the scaffold. To note, the differentiated cardiomyocytes don’t proliferate. In order to create more tissue layers in the electrode, a higher initial cell number is required. The propagation speed of the electrophysiological signals in cardiac patches of previous works showed speeds of 4 cm/s^12^ and a maximum of 69 cm/s in human native tissue^23^. The fusion of cardiac tissue and electrode resulted in an increased propagation speed over all measuring electrodes. The reason for this behavior may be the abridgment of the signal conduction from the cells into the electrode. The rapid distribution in the electrode supports the simultaneous signal propagation from the electrode into the complete, which supports synchronization.

After the formation of a hybrid electrode, the next step was to test its applicability as a pacemaker electrode. The disadvantage of current 2D pacemaker electrodes is the incompatibility for tissue integration^7^. Although the hybrid 3D electrode already facilitated tissue integration, a seamless connection to the patients’ cardiac tissue still has to be established. Due to the human-based approach, animal models are inappropriate. Therefore, a previously developed cardiac tissue model was applied. After 4 weeks of culture, a unit of cardiac tissue and hybrid electrode was formed without any additional mechanical support measures. The in growth resulted in a mechanically stable interface. Additionally, external pacing of the cardiac muscle was feasible through the embedded leads, covering the physiological frequencies between 1 and 3 Hz. Nevertheless, a decreased contraction force was detected for higher pacing frequencies, which indicates an incomplete regeneration of the electrophysiological cycles for frequencies higher than 1.4 Hz. Compared to highly matured cardiac cells with an increasing force-frequency relationship, hiPS-CM have a reduced calcium diffusion, a slowed calcium accumulation as well as inefficient ATP deployment and diffusion in the cells^24–26^. By applying specialized training protocols during tissue culture, optimized assembly of the mitochondria and accumulation of L-type calcium channels near the sarcomeres can be achieved to generate a highly matured cardiac tissue^27^. Nevertheless, to show the generation and functionality of a hybrid pacemaker, it was not necessarily required to generate cardiac tissue exhibiting this high maturation grade. During the ingrowth of hybrid electrode into the cardiac patch and in the absence of external electrical stimulation, a synchronization between the two components was detected, indicating that at least at the boundary of the electrode, the cells of the hybrid electrode and the patch established physiological electrical connections. To confirm this, further electrophysiological measurements and stainings are required. Interestingly, compared to the power consumption of implanted pacemakers in the range of 1–100 µW^28^, the required pulse power of 5 µW for the stimulated contraction in the *in vitro* construct was near the minimum level of current pacemaker electrodes^28^. Due to the cell free back side of the hybrid electrode and the low impedance towards water based electrolytes, leakage currents are released into the medium and increase thereby the energy consumption of the developed hybrid electrode. Therefore, an initial seeding of the cells on both sides of the scaffold might improve the electrical efficacy and may shift the consumption below the level of current implanted pacemaker. Additionally, the higher initial seeding number of cardiomyocytes would foster the formation of cardiac tissue in deeper regions of the scaffold as well as the integration of the hybrid electrode into the cardiac muscle. Nevertheless, the development of new pacemaker electrodes and electrode materials only lead to an initial and marginal improvement of the stimulation efficiency. But the improvement of the electrode integration and associated significant reduction of the foreign body reaction may improve the stimulation efficiency over the complete lifetime of the electrode.

For a clinical translation, animal models are required. Critical investigations regarding the mechanical long-term stability and safety of the carbon nano fibers are essential. Regarding macrophages, a reduced reaction was detected when comparing 2D and 3D electrodes. However, the foreign body response *in vivo* is still a required criterion. Therefore, a suitable animal model is essential as well as respective cell lines like a porcine induced pluripotent stem cell line and methods for cardiac differentiation. Alternatively, future developments of cardiac hybrid electrodes should also consider whether hdF or hMSC are sufficient to minimize the foreign body response and fibrosis and to foster tissue ingrowth.

## Conclusions

In general, advantages of a 3D carbon nano fiber electrode compared to a 2D TiN electrode were presented. Beside the improved electrical properties, the 3D electrode allowed cell migration into the scaffold. Porosity and cell migration are important criteria for all future implants to support long term compatibility^7^. Additionally, cell migration led to the formation of a hybrid electrode composed of cardiac tissue embedded carbon nano fibers. The fusion of a cardiac tissue model with the hybrid electrode proved the feasibility of a 3D scaffold as cardiac pacemaker electrode that seamlessly integrates into the host’s tissue. Nevertheless, suitable animal models are essential for future integration tests and functional characterizations of a hybrid electrode.

## Electronic supplementary material

Supplementary Video V3

Supplementary Video V4

Supplementary Figures

Supplementary Video V1

Supplementary Video V2
