# Supplementary material for: A three-dimensional hybrid pacemaker electrode seamlessly integrates into engineered, functional human cardiac tissue in vitro
Source: Sci Rep. 2018 Sep 28;8:14545. doi: 10.1038/s41598-018-32790-8 (PMC6162283; doi:10.1038/s41598-018-32790-8)
Supplement: Supplementary file 3 — Supplementary Figures [file 41598_2018_32790_MOESM3_ESM.docx]

A three-dimensional hybrid pacemaker electrode seamlessly integrates into engineered, functional human cardiac tissue *in vitro*

Tobias Weigel^1^, Tobias Schmitz^1^, Tobias Pfister^2^, Sabine Gaetzner^1^, Maren Jannasch^1^, Reem Al-Hijailan^3^, Sebastian Schürlein^1^, Salwa Suliman^4^, Kamal Mustafa^4^, Jan Hansmann^1,2^*

^1^ T. Weigel, T. Pfister, T. Schmitz, S. Gaetzner, M. Jannasch, S. Schürlein, J. Hansmann

University Hospital Würzburg, Department Tissue Engineering and Regenerative Medicine

(TERM), Röntgenring 11, 97070 Würzburg, Germany

^2^ Tobias Pfister, Jan Hansmann

Fraunhofer Institute for Silicate Research, Neunerplatz 2, 97082 Würzburg, Germany

^3^ Reem Al-Hijailan

King Faisal Hospital and research center, Cell Biology Department, research center,

P:O Box: 3354 Mbc03, Riyadh: 11211, Saudi Arabia

^4^ Salwa Suliman, Kamal Mustafa

Department of Clinical Dentistry, Center of Clinical Dental Research, University of Bergen, Årstadveien 19, 5009 Bergen, Norway

*Corresponding Author: Jan Hansmann

University Hospital Würzburg, Department Tissue Engineering and Regenerative Medicine

(TERM), Röntgenring 11, 97070 Würzburg, Germany

E-mail: Jan.Hansmann@uni-wuerzburg.de

Phone: +49 931 31-81209

Fax: +49 931 31-81068


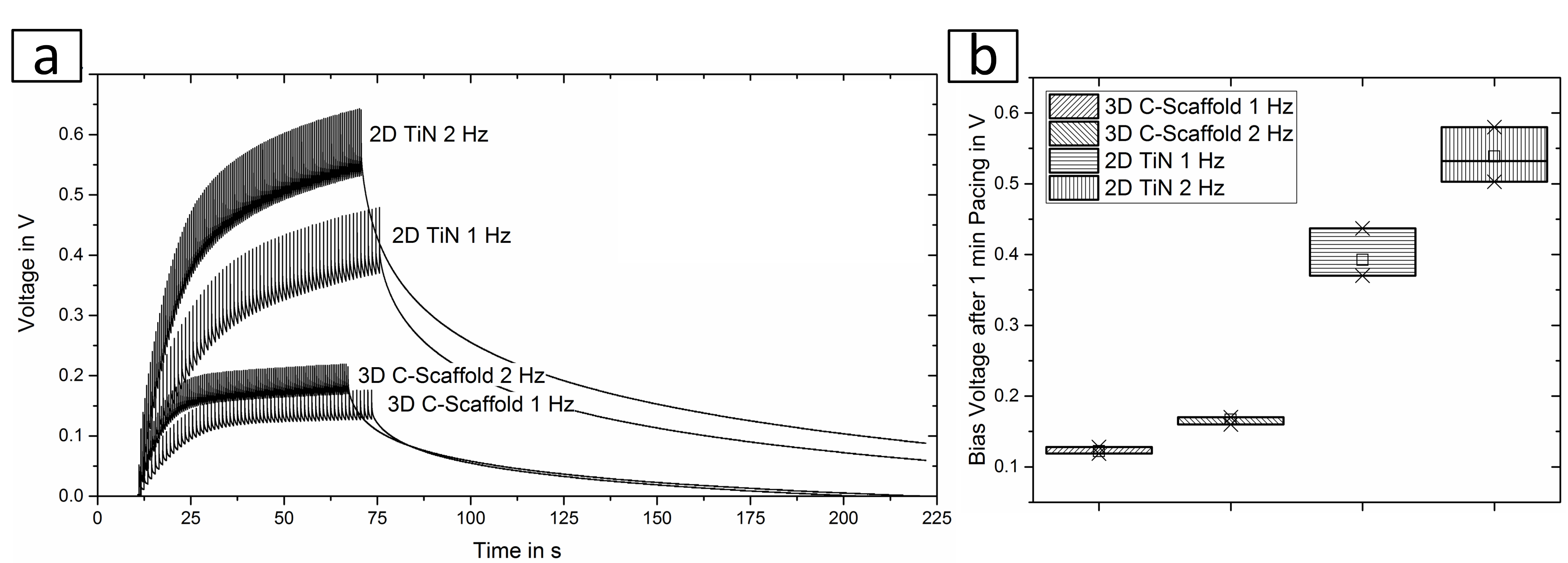


**Supplementary Figure S1. Formation of a bias voltage on the 3D carbon fiber scaffold and the 2D TiN electrode during a continuous pacing process of 1 min.** (a) During a pacing process of the electrode materials into DMEM, the formed voltage was recorded. The figure shows exemplarily one measurement of each material with pacing frequencies of 1 and 2 Hz and the discharging in the following 2.5 min. (b) Boxplot of the formed bias voltage after 1 min of pacing (n=3).


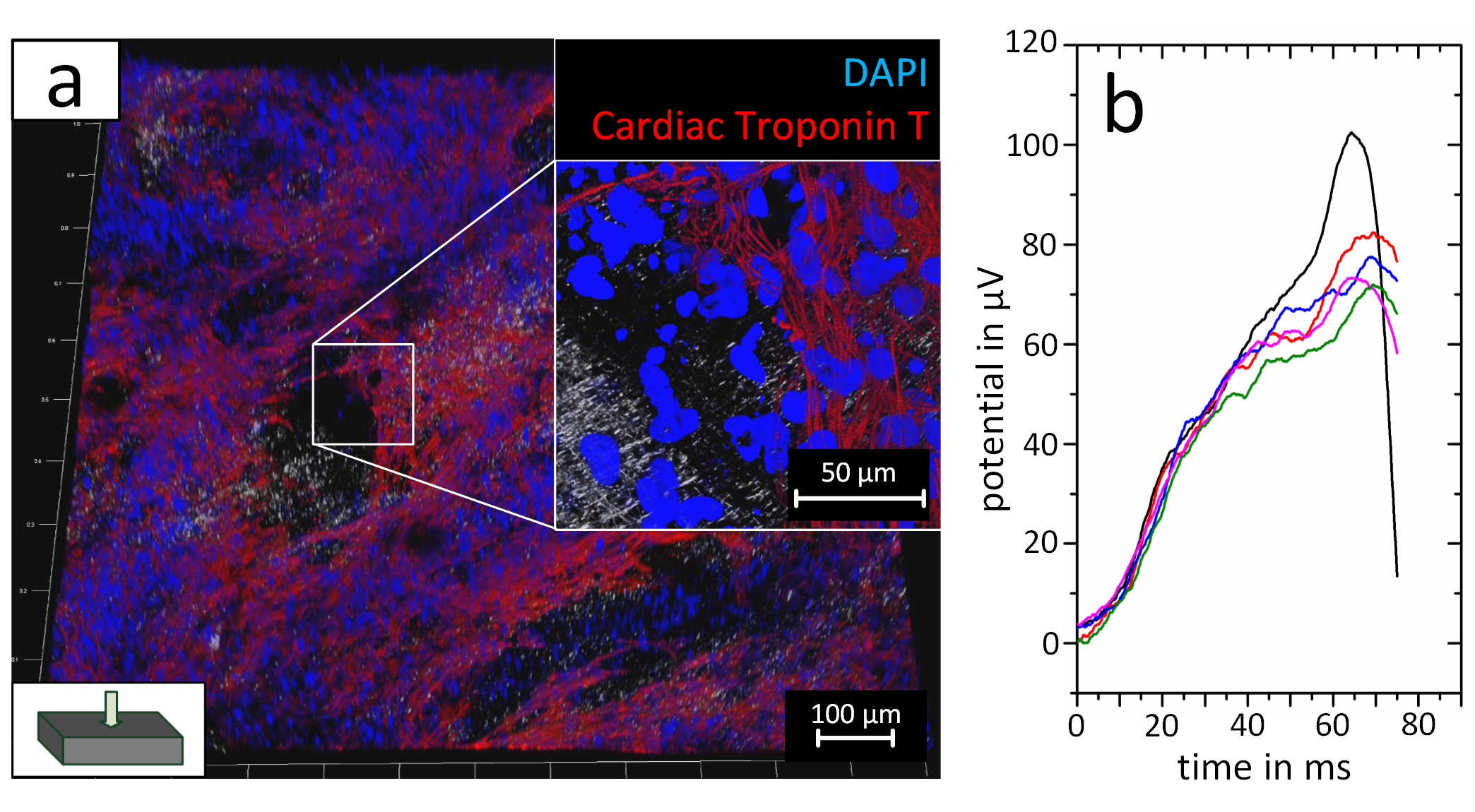


**Supplementary Figure S2. Enhanced details of the hybrid cardiac electrode.** (a) Immunoflourescent staining against cardiac Troponin T (red) on top of the hybrid cardiac electrode. Nuclei are displayed in blue and the fiber electrode in gray. The image shows an overview of around 1.5 mm² and a higher magnified insert. (b) Enlargement of one peak of the MEA in Fig. 4C as an example for the shifted peak maxima in the range of a few ms.


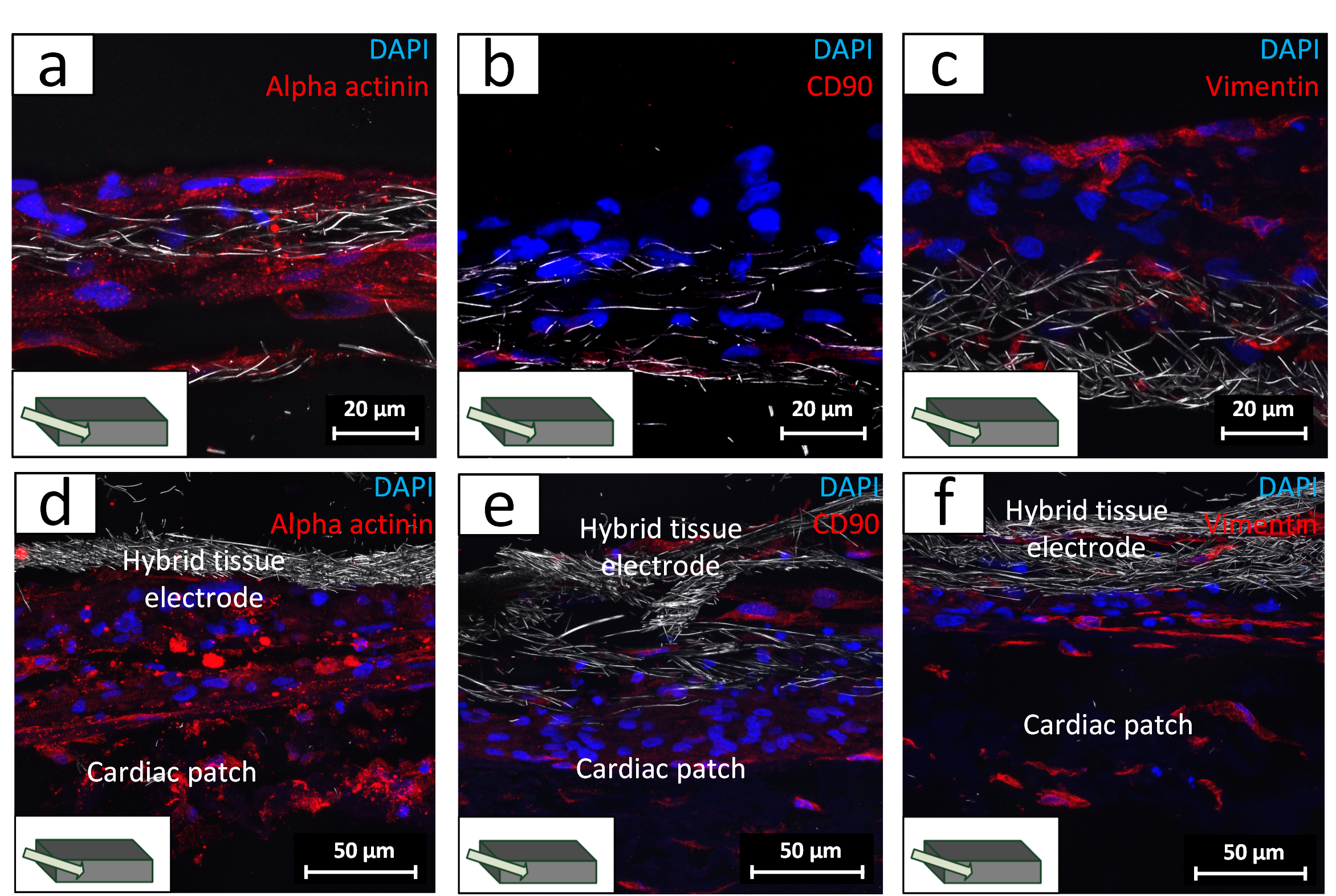


**Supplementary Figure S3. Analysis of the hybrid electrode and the fused electrode/tissue by immunofluorescent staning of the cross sections.** (a-c) Cross sections of the hybrid electrode after 4 weeks of culture. (d-f) Cross sections of the ingrown hybrid electrode and cardiac muscle. The sections are stained against (a,d) alpha actinin, (b,e) CD90 and (c,f) Vimentin. The respective immunoflourescent staining is depicted in red, nuclei in blue (Dapi) and the electrode fibers in gray.
